# Functional Characterization of the Oat (*Avena sativa* L.) TCP Transcription Factor AsTCP38 Reveals Its Role in Low-Nitrogen Stress Tolerance

**DOI:** 10.3390/ijms27177822

**Published:** 2026-08-31

**Authors:** Jing Pan, Zeliang Ju, Xiang Ma, Lianxue Duan, Zhifeng Jia

**Affiliations:** Key Laboratory of Superior Forage Germplasm in the Qinghai-Tibetan Plateau, Qinghai Academy of Animal Husbandry and Veterinary Sciences, Qinghai University, Xining 810016, China; jingpanoffice@qhu.edu.cn (J.P.); juzliang@126.com (Z.J.); qhmky_mx@163.com (X.M.); dlx1234560201@126.com (L.D.)

**Keywords:** *Avena sativa*, overexpression, antioxidant enzyme activity, nitrogen assimilation, protein–protein interactions

## Abstract

Nitrogen limitation restricts plant growth, development, and yield in crops and forage species. Although TCP transcription factors are implicated in diverse abiotic-stress responses, the functions of most *TCP* genes in oat remain unclear. Here, we cloned and characterized the *AsTCP38* gene, which is 1215 bp long and encodes a 405-amino-acid protein. The predicted protein contains a conserved TCP domain and shares its highest sequence similarity with *Arabidopsis thaliana* (*A. thaliana*) *AtTCP15*. The AsTCP38 protein localized to the nucleus, and promoter analysis identified cis-elements associated with light, hormone, and stress responses. We generated *AsTCP38*-overexpressing *A. thaliana* and wheat plants and screened an oat leaf yeast cDNA library for candidate interacting proteins. In these heterologous overexpression lines, *AsTCP38* overexpression was associated with greater abscisic acid (ABA) sensitivity and improved seedling growth under low-nitrogen conditions. Changes in antioxidant-enzyme activities, nitrogen-metabolism-related enzyme activities, and endogenous hormone contents were also observed. Together, these findings suggest that *AsTCP38* may participate in low-nitrogen responses and provide a basis for further functional studies in oat. Direct regulatory targets and the contribution of *AsTCP38* to low-nitrogen adaptation in oat remain to be established.

## 1. Introduction

Oat (*Avena sativa* L.), belonging to the genus Avena of the Poaceae family, is a dual-purpose crop for both food and feed. They are renowned for its excellent drought resistance, cold tolerance, and ability to thrive in poor soil conditions, and has been widely cultivated and promoted worldwide [1]. The growth and development of *Avena sativa* are closely tied to various environmental factors. Among these factors, abnormal temperature, water deficiency, soil salinization, insufficient nitrogen supply, infection by pathogenic microorganisms, and other biological and abiotic stressors can significantly impact the yield and quality of *Avena sativa* [2]. Nitrogen is a vital element for plant growth and development, playing a crucial role in agriculture [3]. However, the global efficiency of nitrogen fertilizer use is less than 50% [4]. As the population grows, the demand for nitrogen fertilizers in farming increases, leading to more nitrous oxide emissions and contributing to global warming [5]. Efficient nitrogen utilization relies on the coordinated regulation of nitrogen absorption and assimilation processes with carbon metabolism, enabling plants to achieve a balance between nutrient acquisition and growth [6]. Improving the nitrogen use efficiency of *Avena sativa* and developing varieties that tolerate low nitrogen levels are important steps toward sustainable grassland agriculture. When plants are subjected to stress, they activate a series of gene expression programs that regulate their tolerance to unfavorable conditions. Plant hormones, such as gibberellin (GA), abscisic acid (ABA), auxin (IAA), and jasmonic acid (JA), play a crucial regulatory role in the process of plants coping with adverse stress [7,8]. Auxin plays a crucial role in nitrogen signal transduction and root development, and thus is regarded as a potential target for improving the nitrogen utilization efficiency of crops [9]. Studies have shown that transcription factors can activate or inhibit the transcriptional activity of target genes by binding to specific cis-acting elements in the promoters of target genes, thereby mediating a response to adverse stress [10]. Members of the TCP transcription factor family in *Avena sativa* play a key role in the regulatory mechanisms that respond to abiotic stress. However, the specific functions of the family members remain to be analyzed in depth. As a result, the study of the TCP transcription factor family in *Avena sativa* has significant biological value and is crucial for understanding the molecular mechanisms underlying plant stress resistance.

The TCP transcription factor family, a group of systemically conserved, plant-specific regulatory factors, is divided into three subfamilies: CINCINNATA (CIN), CYCLOIDEA/TEOSINTE BRANCHED1 (CYC/TB1), and PROLIFERATING CELL FACTOR (PCF), which exhibit significant evolutionary conservation and functional diversity in controlling organ development, growth, and development [11]. Recent studies have further demonstrated that TCP proteins can participate in the construction of complex regulatory networks by integrating upstream signals, protein interactions, and downstream target gene regulation. Thus, they play extensive and crucial roles in plant growth and development as well as in responses to environmental stresses [12]. Genome-wide comparative analysis uncovered interspecies differences in the genomic distribution of TCP members. Twenty-three genes encoding TCP proteins were identified in the dicotyledonous model plant *A. thaliana*, 22 in the monocotyledonous model plant rice (*Oryza sativa*) [13], and 66 in the hexaploid variety wheat (*Triticum aestivum*) [14]. Recent studies on rapeseed have shown that the PCF subfamily has undergone a significant expansion during evolution. Moreover, multiple TCP genes exhibit responsive expression under different stress conditions, further indicating that this family has a rich functional differentiation and regulatory diversity in the process of plant adaptation to the environment [15]. TCP transcription factors can either activate or inhibit the transcriptional activity of downstream stress tolerance-related genes by recognizing and binding to the cis-acting elements in the promoter regions of abiotic stress response genes, thereby regulating the stress response signaling pathways in plants and ultimately improving their ability to adapt to adverse stresses such as drought, high salinity, and extreme conditions [16]. Currently, numerous reports exist on the responses of PCF subfamily genes to abiotic stress [17]. For example, the *OsTCP19* gene in rice significantly enhances plant tolerance to drought and salinity by upregulating the ABA signaling and lipid metabolism pathways during the early stages of stress responses [18].

Meanwhile, integrating stress signals with developmental pathways (such as lateral root formation) plays a vital role in abiotic stress adaptation [19]. Nitrogen-responsive root plasticity is a key factor influencing nitrogen absorption, and the regulation of genes that respond to the root system can enhance the nitrogen utilization efficiency of rice [20]. As a member of the PCF subfamily in rice, *OsTCP21* has been reported to negatively regulate abiotic stress responses, including cold stress, and its expression is significantly induced under cold stress conditions. Overexpression of Os-miR319b suppresses cold-induced *OsTCP21* expression, thereby improving plant tolerance to cold stress through enhanced ROS scavenging, increased proline accumulation, and the activation of cold-responsive genes including DREB1/2 and TPP [21]. This suggests that *OsTCP21* may limit plant stress resistance by inhibiting the stress response pathway. Targeted regulation of its expression can effectively enhance rice’s tolerance to cold stress. As a highly conserved member of the TCP transcription factor family, *BpTCP20* has been reported to alleviate oxidative damage in birch under drought and salinity stresses by enhancing antioxidant enzyme activities, upregulating stress-related genes, and promoting chlorophyll accumulation, thereby improving plant tolerance to drought and salinity. The interaction of *BpTCP20* with *BpMYB8* and *BpIAA5* suggest that it may regulate abiotic stress responses through hormone signaling and antioxidant defense pathways, providing potential targets for improving plant stress tolerance [22]. PCF subfamily genes regulate plant adaptation to abiotic stresses, such as drought and salinity, by modulating hormone signaling pathways, including abscisic acid, gibberellin, and ethylene, and by integrating developmental regulation with stress response mechanisms to enhance plant stress tolerance [23]. For example, *StTCP23*, a member of the PCF subfamily in potato, has been suggested to regulate stress-related hormone signaling by balancing growth and defense responses, thereby contributing to plant adaptation under adverse conditions [24].

Under high-temperature induction, TCP transcription factors *TCP14* and *TCP15* enhance cell elongation by regulating the SMALL AUXIN UP RNA (*SAUR*) genes, including *SAUR63* and auxin response genes (such as *IAA19*). At the same time, the loss of their functions leads to a decrease in SAUR expression and defects in hypocotyl elongation [25]. Taken together, studies suggest that the PCF subfamily of TCP transcription factors contributes to plant responses to both biotic and abiotic stresses. The PCF subfamily is the largest TCP transcription factor family in *Avena sativa*, and its members are closely associated with abiotic stress responses. However, the functions of many PCF subfamily members in *Avena sativa* remain unknown. Previously, the *Avena sativa TCP* gene *AsTCP38* was identified through genome-wide analysis and found to be associated with low-nitrogen stress responses [26]. Recent studies in rice genetics have shown that genetic variations in transcriptional regulatory factors can significantly affect the absorption and utilization efficiency of nitrate. This further highlights the importance of identifying and analyzing crop nitrogen-responsive transcription factors in revealing nitrogen adaptation mechanisms and improving nitrogen utilization efficiency [27]. An in-depth study on the response mechanism of the *AsTCP38* gene in *Avena sativa* under abiotic stress conditions is conducive to revealing its biological functions and providing a scientific basis for clarifying its regulatory pathways.

Although previous studies have shown that TCP transcription factors are involved in plant growth and development, as well as in responses to various abiotic stresses, their specific regulatory roles in *Avena sativa* adaptation to low-nitrogen conditions remain poorly understood. *AsTCP38* has been identified as a member of the *Avena sativa* TCP family; however, its subcellular localization, transcriptional response to nitrogen limitation, contribution to low-nitrogen adaptation, and potential protein-interaction network have not been sufficiently validated experimentally. Nitrogen deficiency is an important constraint on crop growth, yield formation, and nitrogen-use efficiency. Identifying and characterizing the regulatory genes involved in low-nitrogen responses is therefore important for understanding the mechanisms underlying nitrogen adaptation in *Avena sativa* and for identifying potential targets for genetic improvement. Accordingly, we combined sequence and promoter cis-element analyses, subcellular localization, heterologous overexpression in *A. thaliana* and wheat, physiological and hormonal measurements under low-nitrogen conditions, and protein-interaction screening to investigate the function of *AsTCP38*. Specifically, we evaluated whether *AsTCP38* contributes to plant adaptation to low nitrogen and examined the physiological pathways and candidate interacting proteins associated with this response. These research results have further expanded our understanding of the role of TCP transcription factors in the nitrogen stress response, and indicate that *AsTCP38* may act as a potential positive regulatory factor in the low-nitrogen adaptation process of *Avena sativa*.

## 2. Results

### 2.1. Bioinformatics Analysis of the AsTCP38 Protein

**Figure 1:**
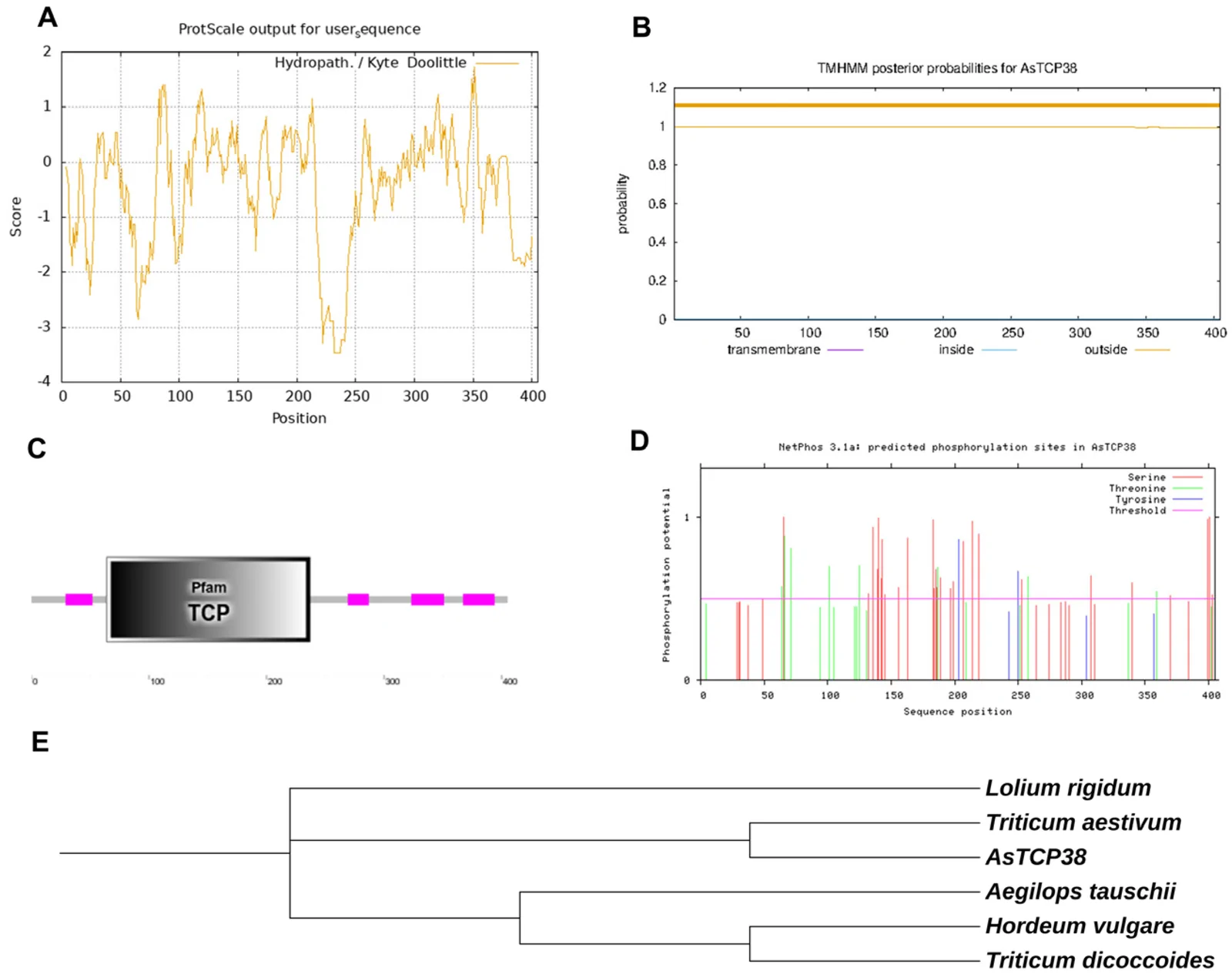
Bioinformatics analysis of *AsTCP38*. (**A**) Hydrophilic/hydrophobic prediction; (**B**) transmembrane structure prediction; (**C**) protein domain prediction; (**D**) phosphorylation site prediction. (**E**) Phylogenetic tree analysis of *AsTCP38* and five species.

The physicochemical properties of the AsTCP38 protein were predicted (Table 1). The relative molecular mass of the AsTCP38 protein is 40,743.01 Da. Among them, the isoelectric point of the AsTCP38 protein is greater than 7, which is an alkaline protein, and the instability coefficient is 63.08, which is an unstable protein. The hydrophilic coefficient of the protein is −0.469, which is a hydrophilic protein (Figure 1A). The aliphatic index is the relative content of the fat side chains in a protein, which is determined by the contents of alanine (Ala), valine (Val), isoleucine (Ile), and leucine (Leu) in the protein. The lipid coefficient of the AsTCP38 protein is 57.26, indicating that the content of aliphatic amino acids in the AsTCP38 protein is high. Analysis with the TMHMM online software revealed that the AsTCP38 protein lacks a transmembrane domain, indicating that AsTCP38 is not a transmembrane protein (Figure 1B). The analysis results of the Smart online software suggest that the 64–237 amino acids encoded by this gene form a conserved TCP domain and belong to a typical TCP transcription factor family. The protein structure encoded by this gene is shown in Figure 1C. Subcellular localization analysis indicated that the *AsTCP38* gene is localized to the cell nucleus. Transcription factors are frequently phosphorylated by protein kinases. Bioinformatic analysis identified 37 putative phosphorylation sites in AsTCP38, including eight threonine (Thr), two tyrosine (Tyr), and 27 serine (Ser) phosphorylation sites (Figure 1D). These finding suggests that AsTCP38 may undergo phosphorylation by protein kinases at these sites, which may modulate its transcriptional activity and influence downstream gene expression.

The protein sequences encoded by *AsTCP38* were compared using the Blastp function on the NCBI website. Five homologous sequences with the highest similarity were selected, and sequence alignment analysis was performed using DNAMAN software v10.0 (Figure S1). As shown in Figure 1E, the *AsTCP38* gene of *A. sativa* has the highest homology and the closest evolutionary relationship with wheat (*T. aestivum*).

### 2.2. Cis-Acting Elements and Subcellular Localization of the AsTCP38 Gene

The cis-acting elements of the *AsTCP38* gene promoter were analyzed using the PlantCARE online tool (http://bioinformatics.psb.ugent.be/webtools/plantcare/html/ (accessed on 14 December 2024)). The results (Table 2) show that there are 16 photoresponsive cis-acting elements (G-box, TCT-motif, I-box, GT1-motif, MRE, etc.) in its promoter region, including 1 SA response element (TCA-element), 3 GA response elements (P-box), and two abiotic stress response elements (DRE). This suggests that the *AsTCP38* gene may be involved in responding to abiotic stress and various hormone signaling pathways.

**Figure 2:**
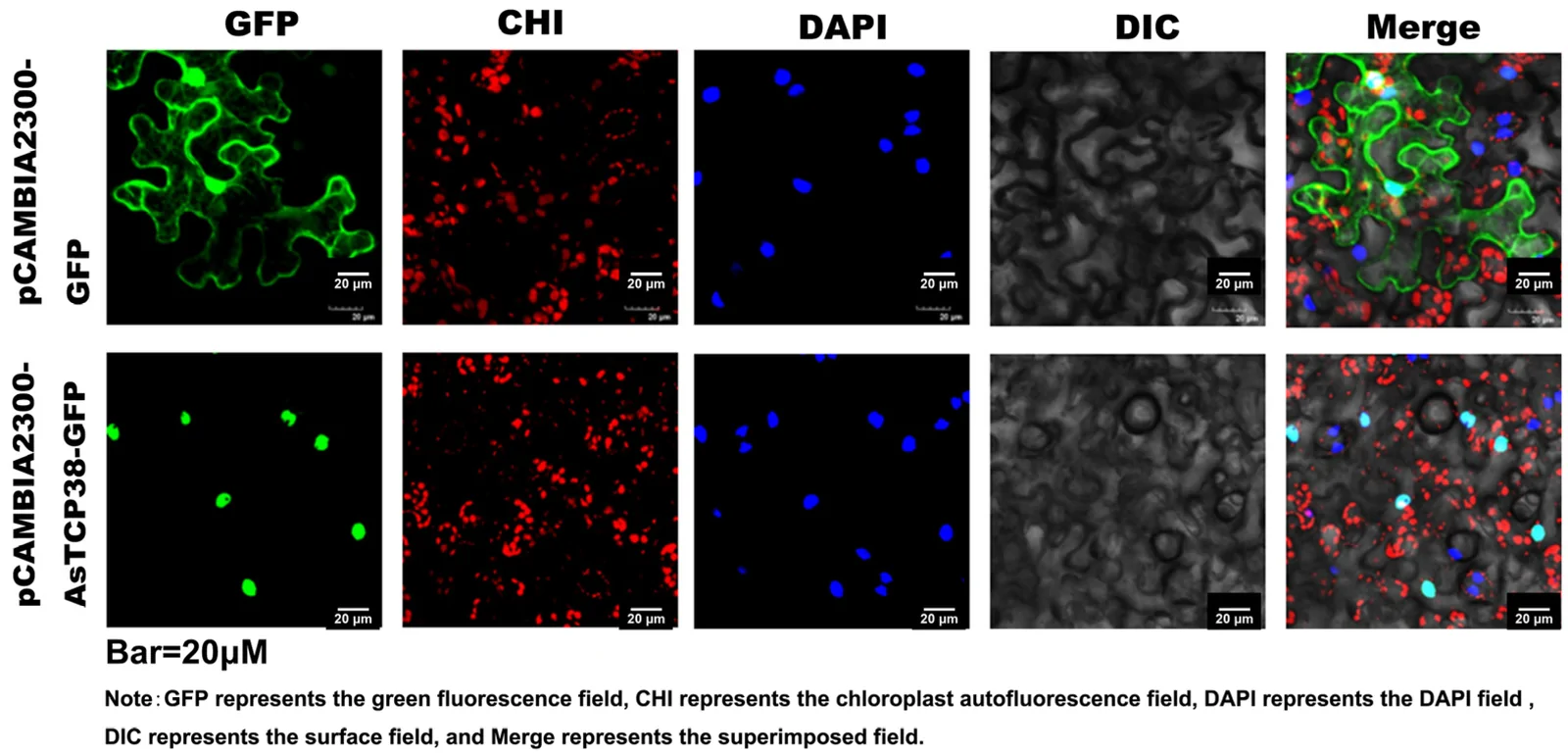
Subcellular localization of AsTCP38 in *Nicotiana benthamiana* leaves.

The prediction results of the cell-PLOC 2.0 online software indicated that the *AsTCP38* gene was in the Cell nucleus. To further investigate the subcellular localization of the AsTCP38 protein, the pCAMBIA2300-*AsTCP38*-GFP vector was constructed. The empty plasmids of pCAMBIA2300-*AsTCP38*-GFP and pCAMBIA2300-GFP were transformed into the Agrobacterium strain GV3100 and then injected into *Nicotiana benthamiana* leaves. The results of microscopic subcellular localization are shown in Figure 2. Green fluorescence was only seen in the nuclei of *Nicotiana benthamiana* mesophyll cells in pCAMBIA2300-*AsTCP38*-GFP. The above results show that the AsTCP38 protein is in the cell nucleus and functions as a typical nuclear transcription factor.

### 2.3. Cloning of the AsTCP38 Gene

The target fragment was cloned using the cDNA of *Avena sativa* leaves as a template, and an amplified fragment of approximately 1200 bp was obtained, which was consistent with the theoretical length of 1215 bp of the product amplified by specific primers (Table S1). The target band was excised from the agarose gel, purified, and transformed into *Escherichia coli*. Positive clones were identified by colony PCR and selected for sequencing (Figure S2). The sequencing results showed that AsTCP38 encodes a protein of 405 amino acids, confirming the successful cloning of the AsTCP38 coding sequence for subsequent construction of the plant expression vector.

### 2.4. Development of Transgenic A. thaliana and Wheat with Overexpression of AsTCP38

**Figure 3:**
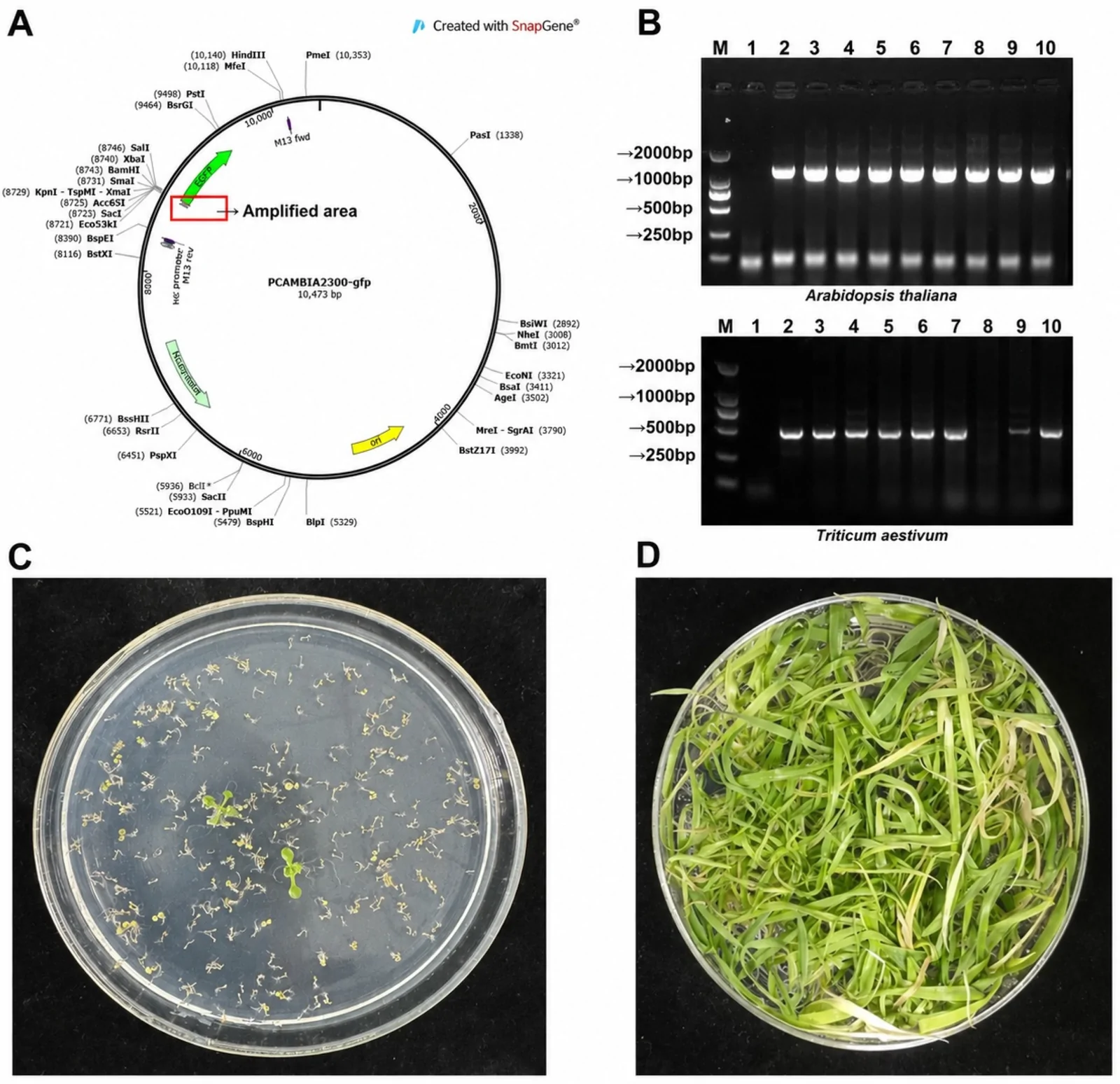
Establishment and molecular identification of the *AsTCP38* overexpression system. (**A**) Schematic diagram of the pCAMBIA2300-GFP plant expression vector. (**B**) PCR detection of the AsTCP38 transgene in putative transgenic plants. (**C**) Screening of positive seedlings of overexpressed *A. thaliana*. (**D**) Screening of overexpressed positive *T. aestivum* seedlings.

The plant expression vector pCAMBIA2300-AsTCP38-GFP was introduced into the *Agrobacterium* tumefaciens GV3101 (Figure 3A), and *A. thaliana* (Col-0) and wheat (field) were subsequently transformed using the floral-dip method and biolistic transformation, respectively. Following hygromycin selection, genomic PCR confirmed the presence of the exogenous gene in both transgenic A. thaliana and wheat plants, as indicated by the detection of the expected target bands (Figure 3B). The transgenic *A. thaliana* plant showed higher survival rates on the selective medium and developed normally, with true leaves emerging after germination. In contrast, the wild-type plants developed yellow cotyledons and eventually died under the same selection conditions. In the wheat transformation system, the most resistant shoots successfully developed into complete plants after being transferred to rooting medium containing plant growth regulators (Figure 3D). Transgenic *A. thaliana* and wheat lines with stable agronomic traits and confirmed by molecular analysis were subsequently selected for physiological analyses.

### 2.5. Root Growth and Biomass of AsTCP38-Expressing Arabidopsis and Wheat Under Low-Nitrogen Treatment

**Figure 4:**
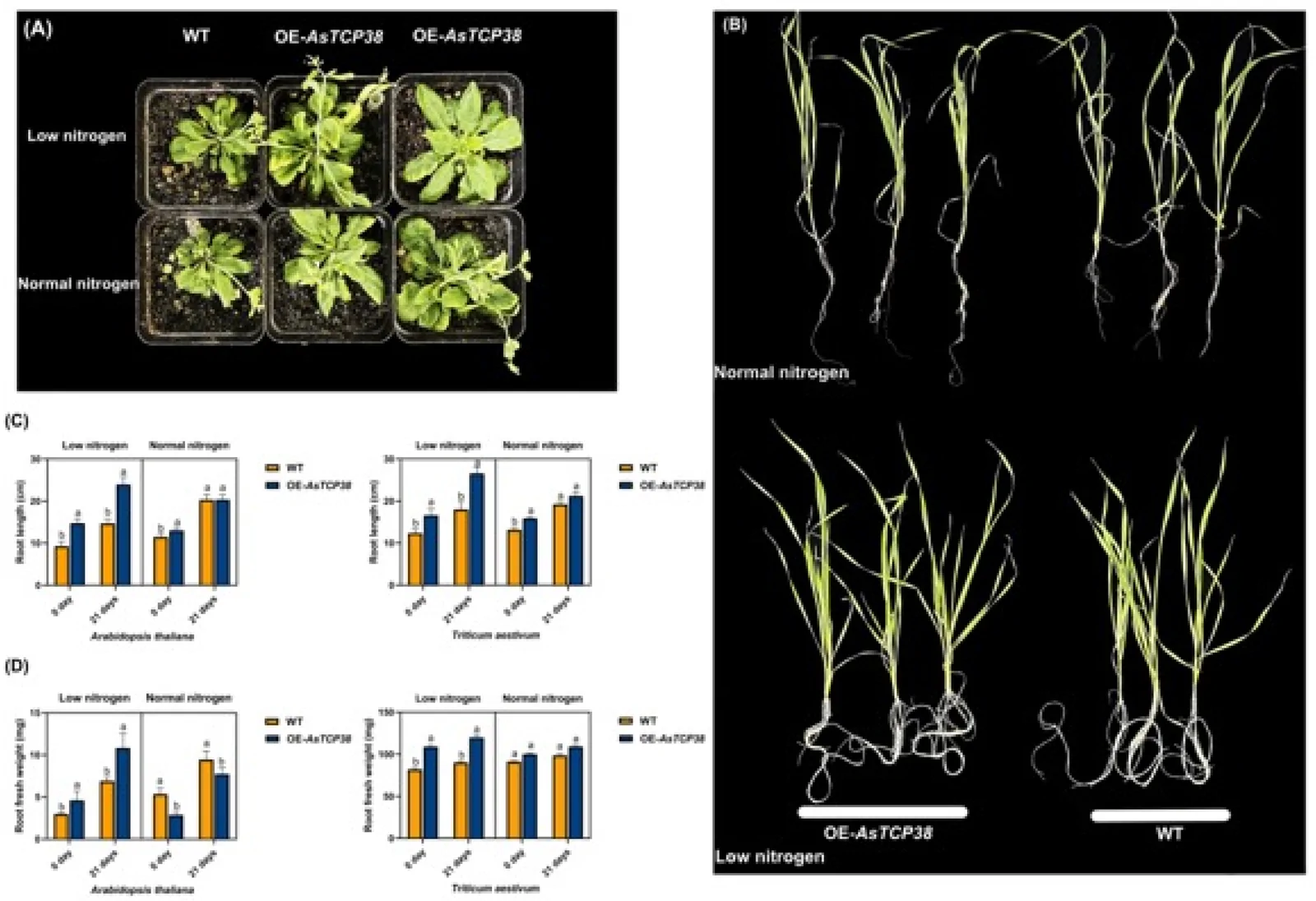
Root responses of OE-*AsTCP38* and wild-type (WT) plants to low-nitrogen treatment. (**A**) Growth phenotypes of *A. thaliana* plants under normal- and low-nitrogen conditions. (**B**) Growth phenotypes of *T. aestivum* plants under normal- and low-nitrogen conditions. (**C**) Root length measured at 0 and 9 days after the initiation of the treatments. (**D**) Root fresh weight measured at 0 and 9 days after the initiation of the treatments. Data are shown as the mean ± SE of three independent experimental units (*n* = 3), and error bars represent SE. Two-factor analysis of variance (ANOVA) was conducted separately for the data of different sampling times for each species, and the Duncan multiple comparison method was used for the significance test of differences. Lowercase letters indicate significant differences among different genotypes within the same species, sampling time, and nitrogen fertilizer treatment conditions; different letters indicate significant differences (*p* < 0.05), and the same letters indicate non-significant differences (*p* > 0.05).

With the extended low-nitrogen stress period, the growth of both WT and the transgenic *A. thaliana* was affected. The WT grew slowly, but the transgenic *A. thaliana* grew normally (Figure 4A). The root length and fresh weight of the OE-*AsTCP38* and WT *A. thaliana* and wheat (Figure 4B) showed an increasing trend with the duration of low nitrogen stress, but the transgenic lines had significantly higher root length and fresh weight than those of WT (*p* < 0.05). Under low nitrogen stress conditions, transgenic *A. thaliana* (OE-*AsTCP38*) overexpressing the *AsTCP38* gene and wheat plants showed significantly enhanced low nitrogen adaptability: compared with the wild type (WT), the root length and fresh weight of OE plants increased by 38.2% and 25.7% respectively (Figure 4C,D), and the root configuration showed low-nitrogen specific optimization (elongation of the primary root). However, under normal nitrogen conditions, no significant difference in phenotype was observed between OE-*AsTCP38* and the wild-type plants (*p* > 0.05).

### 2.6. AsTCP38 Overexpression Improves Oxidative Stress Tolerance in A. thaliana and Wheat via Modulation of Antioxidant Enzyme Networks

**Figure 5:**
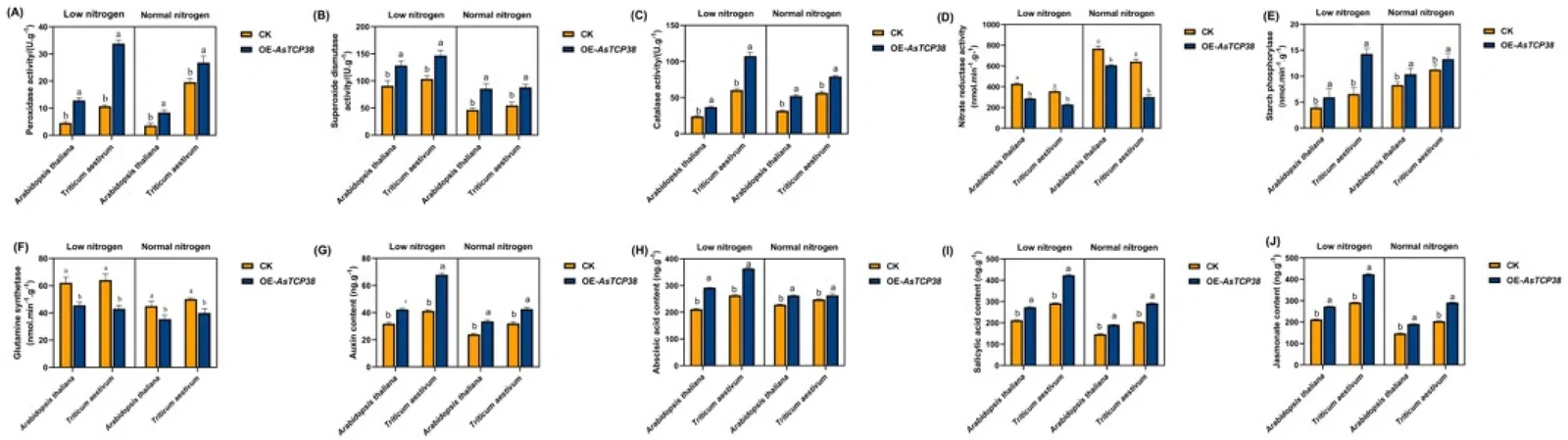
Analysis of physiological indicators in *Arabidopsis* and wheat overexpressing the *AsTCP38* gene. (**A**) Peroxidase activity. (**B**) Superoxide dismutase activity. (**C**) Catalase activity. Note: Different lowercase letters indicate significant differences among the same treatment day (*p* < 0.05, Duncan). The same below. (**D**) Nitrate reductase activity. (**E**) Starch phosphorylase activity. (**F**) Glutamine synthase activity. (**G**) Auxin content. (**H**) Abscisic acid content. (**I**) Salicylic acid content. (**J**) Jasmonic acid content. Data are shown as the mean ± SE of three independently pooled biological samples (*n* = 3), and error bars represent SE. Two-factor analysis of variance (ANOVA) was conducted separately for the data of different sampling times for each species, and the Duncan multiple comparison method was used for the significance test of differences. Lowercase letters indicate significant differences among different genotypes within the same species, sampling time, and nitrogen fertilizer treatment conditions; different letters indicate significant differences (*p* < 0.05), and the same letters indicate non-significant differences (*p* > 0.05).

Under low nitrogen stress, the activities of peroxidase (POD), superoxide dismutase (SOD), and catalase (CAT) progressively increased in transgenic *A. thaliana* and *T. aestivum* plants overexpressing *AsTCP38* (Figure 5A–C). Among the transgenic lines, the increase in antioxidant enzyme activities was greater in the *A. thaliana* OE-*AsTCP38* lines than in the corresponding wheat transgenic lines, suggesting a stronger response to low-nitrogen stress in the dicotyledonous model plant. The increase in antioxidant enzyme activities was also positively associated with the severity of low-nitrogen stress. In contrast, no significant differences in enzyme activities were observed between the transgenic and wild-type plants under normal-nitrogen conditions (*p* > 0.05). These results suggest that the *AsTCP38* gene improves the plant’s resistance to oxidative damage, causing low nitrogen levels by activating the synergistic effect of the reactive oxygen species (ROS) clearance system, suggesting that the function of this gene may be closely linked to the complexity of the antioxidant regulatory network that has evolved in plants.

### 2.7. AsTCP38 Overexpression Improves Low-Nitrogen Adaptation in A. thaliana and Wheat Through Coordinated Regulation of Nitrogen Assimilation and Carbon Metabolic Enzymes

Under low nitrogen stress conditions, the activities of nitrate reductase (NR), glutamine synthase (GS), and starch phosphorylase (SP) in transgenic *A. thaliana* and wheat plants overexpressing *AsTCP38* were notably higher than those in the wild type. Among them, the increase in enzyme activity in *A. thaliana* was considerably greater than that in wheat (Figure 5D–F). Under normal-nitrogen conditions, transgenic *A. thaliana* plants exhibited significantly higher NR activity than the corresponding wild-type plants, whereas transgenic wheat plants showed significantly lower NR activity than their wild-type counterparts. Furthermore, under low nitrogen stress, the activities of nitrogen assimilation enzymes (NR/GS) and carbon metabolism enzymes (SP) in transgenic lines showed a synergistic enhancement trend. However, this synergistic effect was more pronounced in *A. thaliana*, indicating that *AsTCP38*’s regulation of the nitrogen assimilation pathway shows significant species specificity.

### 2.8. Overexpression of AsTCP38 Enhances the Low-Nitrogen Adaptability of A. thaliana and Wheat Through Endogenous Hormone Expression

Under low nitrogen stress conditions, the contents of IAA, ABA, salicylic acid (SA), and JA in transgenic wheat plants overexpressing *AsTCP38* were significantly higher than those in the control group (Figure 5G–J), among which the increases of ABA and SA were the most prominent. Meanwhile, low nitrogen stress significantly induced the synchronous accumulation of similar hormones in transgenic *A. thaliana*, indicating that the gene’s regulatory function is conserved across species. Under normal nitrogen conditions, the contents of IAA and JA in transgenic wheat were lower than those in the wild type. In contrast, the contents of ABA and SA showed no significant changes, indicating that *AsTCP38* has low nitrogen specificity in regulating hormone homeostasis. Further analysis revealed that the coordinated increases in the levels of the four hormones under low-nitrogen stress were significantly associated with increased root biomass, as indicated by higher fresh root weight and enhanced antioxidant enzyme activities in transgenic wheat. These results indicate that the *AsTCP38* gene activates wheat’s stress response by participating in the hormone interaction network under low nitrogen stress (such as the ABA-JA co-signaling pathway), thereby enhancing its adaptability to the low nitrogen environment. However, its functional performance might depend on the nitrogen level in the environment.

### 2.9. Construction of an Avena Sativa Yeast Library Under Low Nitrogen Stress

Under low nitrogen stress, the *Avena sativa* leaf yeast library construction project successfully created a three-level library system with high capacity and integrity. The capacity of the primary library was 1.28 × 107 CFU mL^−1^. The capacities of the secondary libraries in the nuclear and membrane systems reached 1.12 × 10^7^ CFU/mL and 1.36 × 10^7^ CFU/mL, respectively. The recombination rate of all libraries reached 100%, and the average inserted fragment length was >1000 bp. Moreover, the concentration of the yeast working solution (1.50 × 107 cells/mL) met the experimental standards (Figure S3A). The total DNA amounts for the secondary library of the nuclear system (pGADT7-DEST vector) and the secondary library of the membrane system (pPR3-n-DEST vector) reached 559 μg and 843 μg, respectively, which are significantly higher than the primary library’s 304 μg. The results confirmed the successful purification of mRNA, the synthesis of cDNA, and insertion of cDNA fragments during library construction. In particular, the PCR identification of 24 random clones confirmed the correctness of the inserted fragments (Figure S3B). The results indicate that the two-hybrid library of *Avena sativa* leaf yeast, constructed using Gateway technology under low-nitrogen stress, exhibits high coverage and reliability, and can be utilized to systematically analyze the interaction network of *Avena sativa* low-nitrogen stress-responsive proteins.

### 2.10. Screening of Interacting Proteins as per AsTCP38

**Figure 6:**
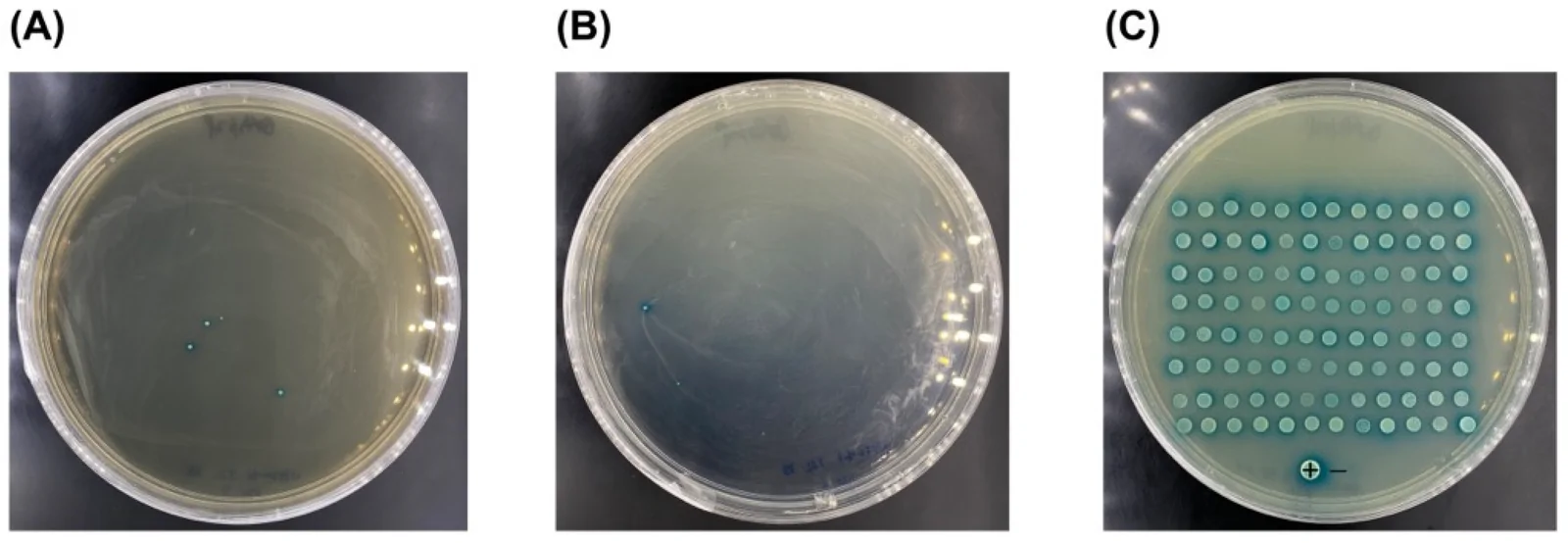
Y2H library screening with AsTCP38 as bait. (**A**,**B**) Representative positive clones from the initial screen on QDO/X/A medium. (**C**) Positive clones after re-screening on QDO/X/A medium.

To identify interacting proteins of AsTCP38, we constructed a BD vector and co-transfected pGBKT7-AsTCP38 with an *Avena sativa* yeast cDNA library for screening. A total of 96 blue clones were found on the QDO/X/A screening plate (partial figures are shown in Figure 6A,B). The blue clones on the primary screening plate were selected and transferred to the QDO/X/A screening plate for re-screening. The results showed that 87 candidate-positive clones grew blue (Figure 6C).

Positive clones were selected for sequencing and subsequent bioinformatic analysis (Table S2). GO functional annotation showed that the candidate interacting proteins identified by the yeast two-hybrid (Y2H) assay were mainly classified into three categories: biological process (BP), cellular component (CC), and molecular function (MF) with 121, 150, and 67 genes, respectively (Figure S4A). KEGG pathway enrichment analysis showed that the candidate interacting proteins were mainly enriched in pathways related to amino acid metabolism, energy metabolism, carbohydrate metabolism, signal transduction, and terpenoid and polyketide metabolism (Figure S4B).

**Figure 7:**
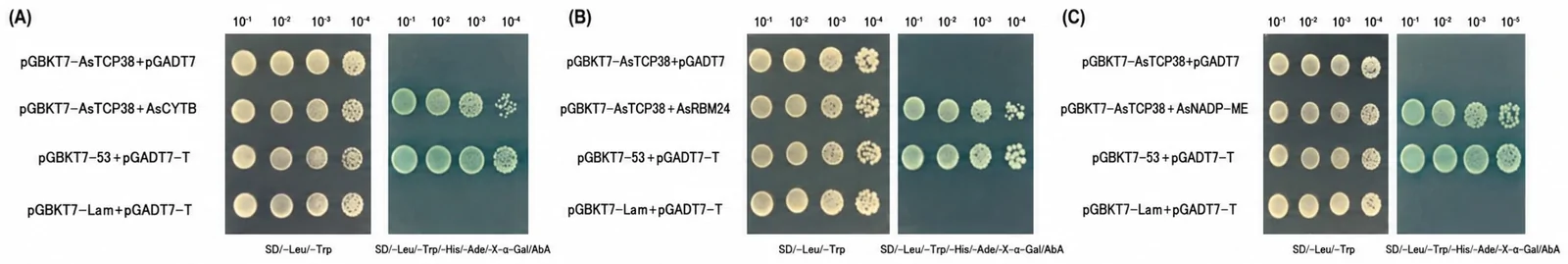
Pairwise Y2H validation of the candidate interactions. (**A**) AsTCP38-AsCYTB; (**B**) AsTCP38-AsRBM24; (**C**) AsTCP38-AsNADP-ME.

**Figure 8:**
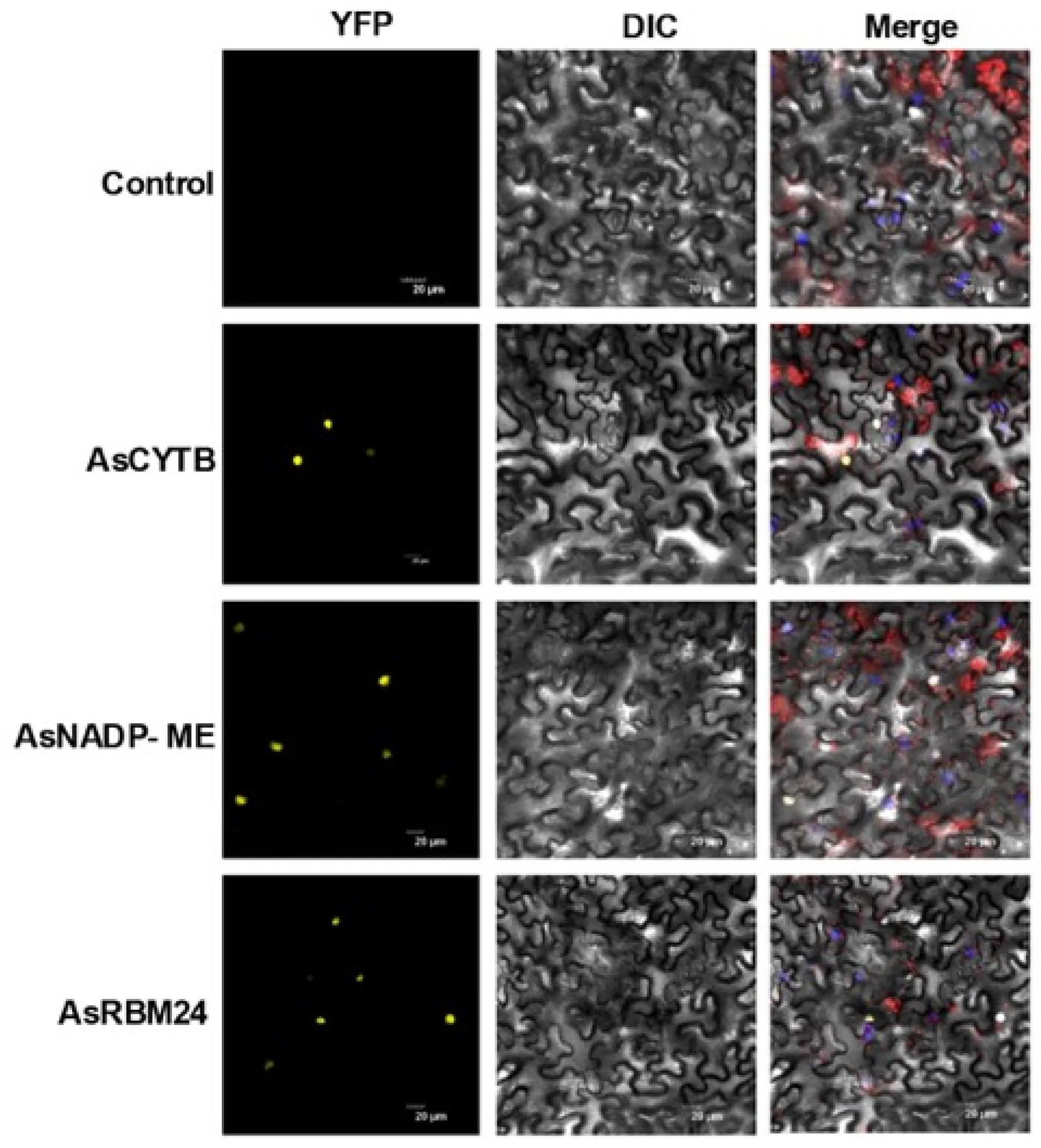
Bimolecular fluorescence complementation analysis of the candidate interactions between AsTCP38 and AsCYTB, AsRBM24 or AsNADP-ME in Nicotiana benthamiana leaves. Images are representative of three independent experiments with consistent results.

Based on the sequencing results, three full-length open reading frames (ORFs) encoding putative AsTCP38-interacting proteins were identified. The corresponding sequences were annotated using the NCBI database. The three candidate interacting proteins were AsCYTB (cytochrome b, GenBank: KAE8813716.1), AsRBM24 (RNA-binding motif protein 24, GenBank: XP_020198048.1), and AsNADP-ME (NADP-dependent malic enzyme, GenBank: XP_010231631.1). In the Y2H assay, AsTCP38 showed no self-activation activity in yeast. The recombinant plasmids and the corresponding positive and negative controls were transformed into Y2HGold yeast cells. Yeast colonies were observed on SD/-Trp/-Leu/-His/-Ade medium (Figure 7A–C). The results indicated that AsTCP38 interacted with AsCYTB, AsRBM24, and AsNADP-ME. To further validate these interactions in planta, a bimolecular fluorescence complementation (BiFC) assay was performed in *Nicotiana benthamiana* leaves. No YFP fluorescence signal was detected in the negative control. In contrast, yellow fluorescence signals were observed in the nuclei of leaves co-transformed with nYFP-AsCYTB, nYFP-AsRBM24, or nYFP-AsNADP-ME, and cYFP-AsTCP38. These signals overlapped with the nuclear staining (Figure 8), supporting the interactions of AsTCP38 with AsCYTB, AsRBM24, and AsNADP-ME in plant cells.

### 2.11. Expression Analysis of AsTCP38 in A. thaliana and Wheat

**Figure 9:**
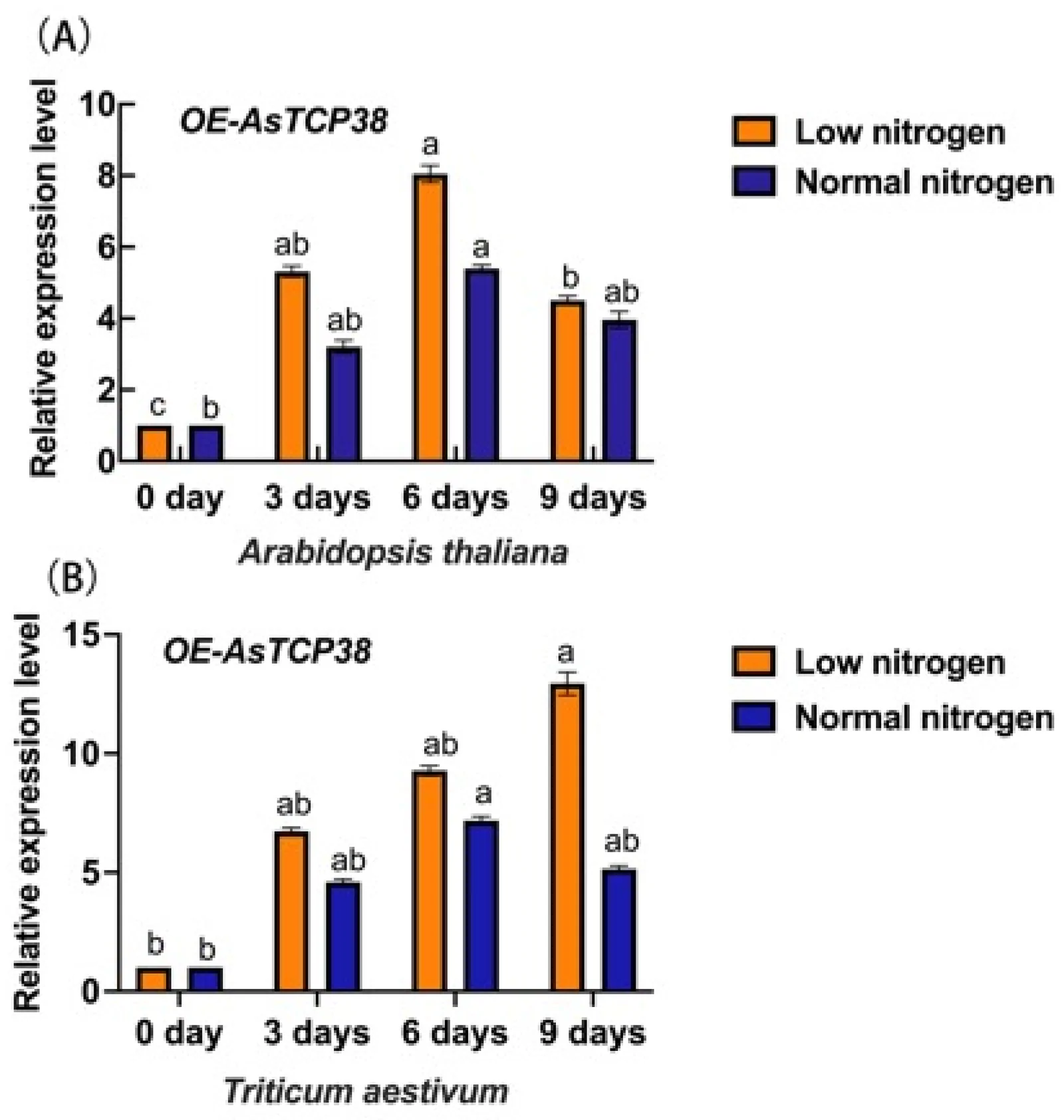
Relative expression of *AsTCP38* in leaves under normal- and low-nitrogen conditions. (**A**) *A. thaliana* and (**B**) *T. aestivum*. Relative transcript levels were determined by qRT-PCR. Three technical reactions were performed for each biological sample, and the resulting values were averaged before statistical analysis. Data are presented as the mean ± SE of three independent biological replicates (*n* = 3), and error bars represent SE. For each species, the effects of nitrogen treatment and sampling time were analyzed by two-way ANOVA, followed by Duncan’s multiple range test. Within the same species and nitrogen treatment, different lowercase letters indicate significant differences among sampling times at *p* < 0.05, whereas values sharing at least one letter are not significantly different.

Total RNA was extracted from the leaves of transgenic *A. thaliana* and wheat overexpressing *AsTCP38*. Following reverse transcription and qRT-PCR analysis, the expression pattern of *AsTCP38* under low-nitrogen stress was examined at different time points. The expression of *AsTCP38* showed a dynamic pattern, with an initial increase followed by a decrease in transgenic *A. thaliana*, whereas its expression in transgenic wheat continued to increase throughout the treatment period (Figure 9). In *A. thaliana* OE-*AsTCP38* plants, *AsTCP38* expression reached its highest level at 6 d, at 7.82-fold that of the corresponding control. In transgenic wheat, *AsTCP38* expression remained elevated at 9 d, reaching 12.93-fold that of the control. These results suggest that *AsTCP38* responds to low-nitrogen stress and may contribute to the regulation of plant responses to nitrogen deficiency.

### 2.12. AsTCP38-Dependent Adaptation to Low-Nitrogen Stress

**Figure 10:**
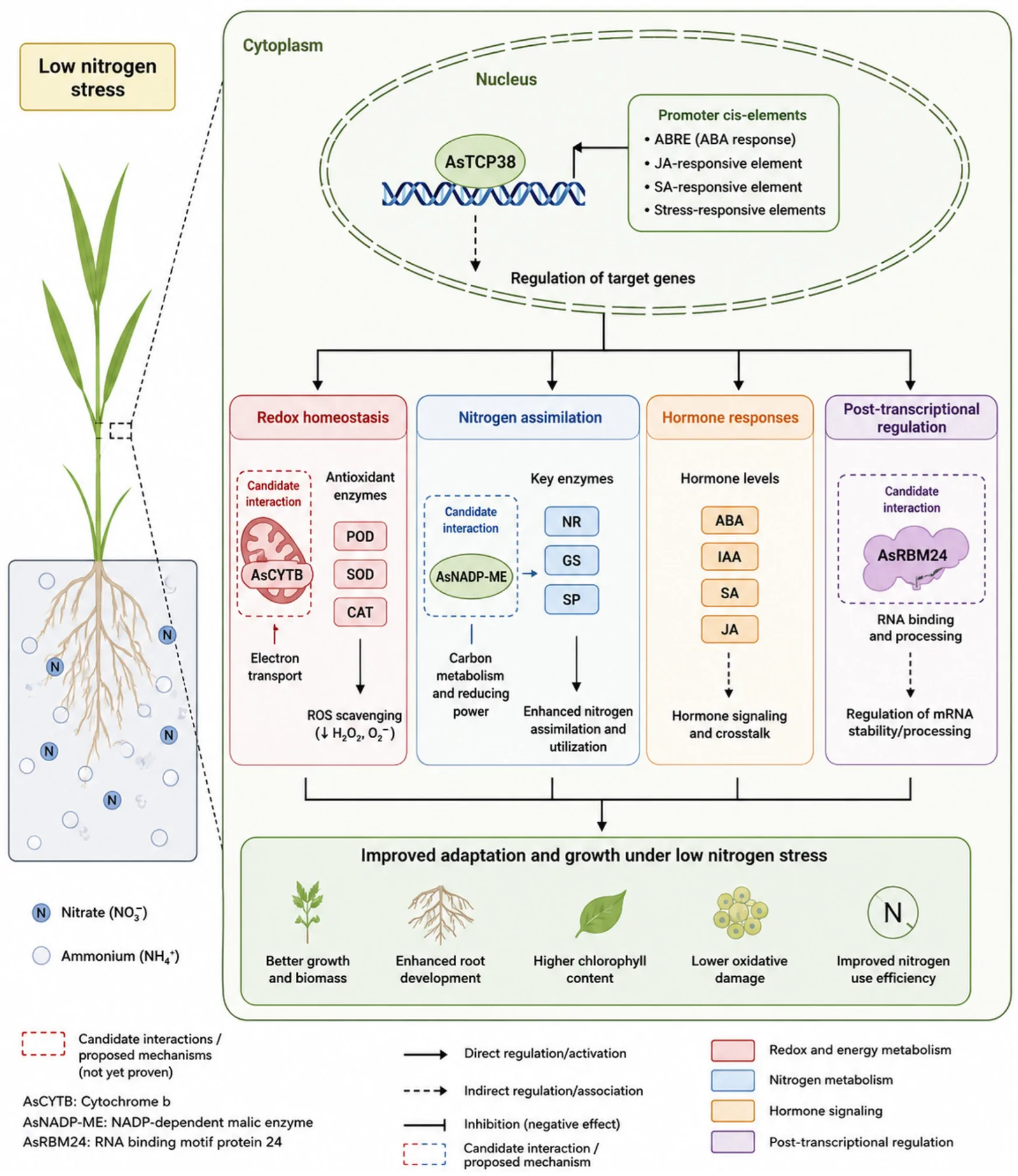
Proposed working model of *AsTCP38*-associated responses to low-nitrogen stress.

Based on the combined expression, protein-interaction, physiological, enzyme-activity and hormone data, we propose a working model for the involvement of *AsTCP38* in the plant response to low-nitrogen stress (Figure 10). The model summarizes the observed association of *AsTCP38* with changes in antioxidant defense, nitrogen-metabolism-related traits and endogenous hormone levels, together with root growth and biomass responses in *AsTCP38*-overexpressing plants. The proteins identified in the yeast two-hybrid screen are shown as candidate components of the *AsTCP38*-associated network. Direct downstream targets and the functional requirement of these candidate interactors remain to be experimentally validated.

## 3. Discussion

Low-nitrogen stress is a major abiotic constraint on plant growth, development, and crop yield [28]. Transcription factors contribute to plant responses to nitrogen limitation by linking environmental signals with changes in gene expression [29]. Among them, plant-specific TCP transcription factors contain a conserved TCP domain and participate in the regulation of organ development, hormone responses, and environmental adaptation through DNA binding and protein interactions [30]. A substantial body of work has examined TCP factors in stress responses, particularly drought and salinity [31,32]. For example, heterologous overexpression of *OsTCP19* in *Arabidopsis* enhanced salt tolerance and was accompanied by changes in ABA signaling and jasmonate biosynthesis [18]. Similarly, *Arabidopsis* plants expressing *CmTCP13* showed improved germination and growth under salt stress, together with altered expression of stress-responsive genes [33]. Although transcriptomic studies indicate that *TCP* genes also respond to nitrogen limitation [26], their contribution to this response remains less well-defined. *Avena sativa* contains 49 TCP-family genes, most of which have not been functionally characterized. In the present study, *AsTCP38* was cloned from *Avena sativa*, and the encoded protein was localized to the nucleus, contained the conserved TCP domain, and showed high sequence similarity to *Arabidopsis AtTCP14* [34]. These results establish the molecular identity and nuclear localization of *AsTCP38*. Together with the phenotypic and physiological responses observed in heterologous overexpression systems, they support the possibility that *AsTCP38* participates in plant responses to low nitrogen. However, the endogenous function of *AsTCP38* in *Avena sativa* remains to be determined. Loss-of-function or gene-silencing studies in *Avena sativa*, combined with the identification of its downstream targets, will be needed to define its contribution to low-nitrogen adaptation.

The expression of many *TCP* genes is low under non-stress conditions but changes in response to salinity, temperature, and drought [35]. The functional consequences of these changes can differ among TCP family members. For example, overexpression of *CnTCP4* in Arabidopsis increased cold sensitivity, consistent with a negative role in the cold response of Chrysanthemum nankingense [36]. Promoters of soybean *TCP* genes also contain cis-regulatory elements associated with low temperature, drought, and ABA responses, indicating that *TCP* expression may be integrated with environmental, hormonal, and circadian signals [37]. In the present study, the AsTCP38 promoter contained several hormone- and stress-responsive cis-regulatory elements [38]. In addition, low-nitrogen treatment of *AsTCP38*-overexpressing Arabidopsis and wheat was accompanied by changes in antioxidant and nitrogen-metabolism enzyme activities and in the measured hormone profiles. These observations are consistent with a hormone-associated response involving *AsTCP38*, but they do not establish direct regulation of the enzymes or hormone pathways examined. The combined data therefore support a working hypothesis in which *AsTCP38* is associated with coordinated changes in redox balance, nitrogen metabolism, and hormone signaling under nitrogen limitation. Testing this hypothesis will require direct target-gene analysis and functional validation in *Avena sativa*.

Plants respond to abiotic stress through coordinated changes in morphology, physiology, and gene expression [39]. WRKY transcription factors provide well-characterized examples of this coordination. Several WRKY proteins influence stress performance through changes in reactive oxygen species (ROS) homeostasis, osmotic adjustment, or hormone signaling [40,41]. For instance, *SbWRKY30* expression increased SOD, POD, and CAT activities in transgenic rice and *Arabidopsis* under drought stress [42], whereas *VvWRKY28* expression was associated with changes in malondialdehyde and proline levels during cold stress [43]. In the present study, AsTCP38-overexpressing Arabidopsis and wheat differed from their controls in root growth and biomass under low-nitrogen conditions. These phenotypic differences were accompanied by higher activities of several antioxidant and nitrogen-metabolism enzymes. The concordant changes are consistent with improved performance of the overexpression lines under the experimental low-nitrogen treatment and suggest an association between *AsTCP38* expression, redox regulation, and nitrogen metabolism. Nevertheless, the data do not show that *AsTCP38* directly controls these enzymes, nor do heterologous overexpression experiments establish its native function in *Avena sativa*. The results therefore identify *AsTCP38* as a candidate regulator of low-nitrogen responses and provide a basis for testing its function in *Avena sativa* through loss-of-function analysis and the direct examination of downstream targets.

Protein-interaction approaches provide a useful route for identifying components of plant signaling and regulatory networks [44,45,46,47,48,49]. The quality of a yeast library depends on its capacity, redundancy, and insert-size distribution [50]. The low-nitrogen *Avena sativa* cDNA library constructed here had an initial concentration of 1.28 × 10^7^ CFU mL^−1^, insert sizes of 500–1500 bp, and a recombination rate of 100%, supporting its use for interaction screening. Yeast two-hybrid screening identified AsCYTB, AsNADP-ME and AsRBM24 as candidate AsTCP38-interacting proteins, and the interactions were further supported by BiFC. Their annotated functions provide possible biological contexts for these associations: AsCYTB is linked to mitochondrial electron transport and redox balance [51]; AsNADP-ME participates in malate metabolism and reducing-power generation relevant to carbon-nitrogen coordination [52]; and AsRBM24 is an RNA-binding protein with potential roles in post-transcriptional regulation [53]. Similar associations between transcription factors and metabolism-related proteins have been reported under abiotic stress [54,55]. The interaction data obtained here extend the physiological observations by connecting AsTCP38 with proteins involved in energy metabolism, carbon metabolism, and RNA-related processes. They should, however, be interpreted as evidence of candidate physical associations rather than proof that these proteins mediate the low-nitrogen phenotype. Interaction-domain mapping and functional analysis of the individual partners will be required to determine their contributions to the AsTCP38-associated response.

Transcription factors act as activators or repressors within signaling networks that coordinate plant development and stress responses [56,57]. For example, FcMYB3 regulates a ROS-related pathway in Ficus carica [58], whereas *TaWRKY19* negatively regulates wheat resistance to stripe rust through effects on ROS production [59]. Such studies illustrate how transcriptional regulators can be embedded in broader redox and metabolic networks. Previous work linking a cassava NADP-ME family member to transcriptional regulation of photosynthetic efficiency also provides a relevant context for examining associations between metabolic enzymes and transcription factors [60]. In the present study, candidate *AsTCP38*-interacting proteins associated with mitochondrial electron transport, carbon metabolism and RNA-mediated regulation were identified by yeast two-hybrid screening and supported by BiFC. Together with the nuclear localization, promoter analysis, and physiological responses of the overexpression lines, these findings broaden the evidence linking TCP transcription factors to nitrogen limitation. Specifically, *AsTCP38* overexpression in Arabidopsis and wheat was associated with differences in root growth, antioxidant and nitrogen-assimilation enzyme activities, and endogenous hormone levels under low-nitrogen conditions. The data therefore suggest that *AsTCP38* may participate in low-nitrogen responses through coordinated physiological and metabolic changes. The interaction results further identify candidate components of a possible *AsTCP38*-associated network. Because the functional evidence is derived mainly from heterologous overexpression, these findings do not yet define the endogenous role of *AsTCP38* in *Avena sativa* or the necessity of the identified interaction partners. Functional analysis in *Avena sativa*, together with targeted validation of downstream genes and protein interactions, will be needed to resolve these relationships.

## 4. Materials and Methods

### 4.1. AsTCP38 Gene Bioinformatics Analysis

We used the online website Expasy—ProtParam (http://web.expasy.org/protparam (accessed on 14 December 2024)) for analysis of the physical and chemical properties of protein; TMHMM Server (http://www.cbs.dtu.dk/services/TMHMM-2.0/ (accessed on 14 December 2024)) for the analysis of proteins across the membrane area; ProtScale (https://web.expasy.org/protscale/ (accessed on 14 December 2024)) to analyze the hydrophilic and hydrophobic proteins; Cell—PLoc (http://www.csbio.sjtu.edu.cn/bioinf/plant-multi/ (accessed on 14 December 2024)) to analyze protein subcellular localization [54]. Cis-acting element analysis was performed in the 2000 bp upstream region of the gene promoter using PlantCARE (http://www.plant-care.com/ (accessed on 14 December 2024)). The conserved motif of the gene was analyzed using MEME (https://meme-suite.org/meme/ (accessed on 14 December 2024)) and NCBI-CDD (https://www.ncbi.nlm.nih.gov/cdd/ (accessed on 14 December 2024)), which were used to download the *Avena sativa* TCP structure domain analysis results. These results were then used in the TBtools II v2.453 [61] Gene Structure View module to visualize the structure of the conserved motif and the conserved field. Multiple sequence alignment and phylogenetic tree construction were performed using MEGA 7.0, and the phylogenetic tree was constructed using the neighbor-joining (NJ) method [62].

### 4.2. Gene Cloning of the TCP38 Gene in Avena sativa

Gene-specific primers were designed based on the coding sequence (CDS) of *AsTCP38* (Table S1). The cDNA derived from leaves of *Avena sativa* cultivar Jiayan No. 2 was used as the template, and the full-length CDS of AsTCP38 was amplified using high-fidelity DNA polymerase. The seeds of *Avena sativa* cultivar Jiayan No. 2 were obtained from the Qinghai Academy of Animal Husbandry and Veterinary Sciences. The PCR product was purified by agarose gel extraction. For construction of the plant expression vector, pCAMBIA2300-GFP was linearized by double digestion with SacI and XbaI, and the purified AsTCP38 fragment was inserted into the linearized vector using a homologous recombination-based cloning method to generate pCAMBIA2300-AsTCP38-GFP. The recombinant product was transformed into competent Escherichia coli cells and plated on LB agar containing 50 μg/mL kanamycin. After incubation at 37 °C, resistant colonies were selected and screened by colony PCR. Positive clones were subsequently cultured overnight, and plasmid DNA was extracted. The identity and sequence accuracy of the AsTCP38 insert were confirmed by Sanger sequencing (Qingke Biotechnology Co., Ltd., Beijing, China) before the verified recombinant plasmids were used for subsequent experiments.

### 4.3. Subcellular Localization Analysis

The subcellular localization of AsTCP38 was initially predicted using Cell-PLoc 2.0. For experimental validation, the coding sequence of *AsTCP38* was fused with GFP in the pCAMBIA2300-GFP vector to generate pCAMBIA2300-AsTCP38-GFP. The recombinant construct and the empty pCAMBIA2300-GFP vector, used as a control, were separately introduced into *Agrobacterium tumefaciens* and transiently expressed in *Nicotiana benthamiana* leaves by *Agrobacterium*-mediated infiltration. The nuclear localization marker NLS-mCherry was co-expressed to identify the nucleus. After incubation at 22 °C for 48 h, GFP and mCherry fluorescence signals were observed using a Zeiss LSM 880 confocal laser-scanning microscope. The subcellular localization of AsTCP38 was determined based on the distribution of the GFP signal and its co-localization with the NLS-mCherry nuclear marker.

### 4.4. Acquisition of Transgenic Plants Overexpressing AsTCP38

Using the sequence information of the plant expression vector pCAMBIA2300-GFP, seamless cloning primers were designed, with 15–20 bp sequences homologous to the vector added to both ends of the primers. The AsTCP38 coding sequence was amplified using high-fidelity DNA polymerase, and pCAMBIA2300-GFP was linearized by double digestion with SacI and XbaI. The purified AsTCP38 fragment was inserted into the linearized vector using the ClonExpress Ultra Seamless Cloning Kit to generate pCAMBIA2300-AsTCP38-GFP. After sequence verification, the recombinant plasmid was introduced into *Agrobacterium tumefaciens* strain EHA105 using the freeze–thaw method. Positive colonies were selected on LB agar containing 50 μg/mL kanamycin and verified by colony PCR and Sanger sequencing.

For *Arabidopsis* transformation, *A. thaliana* Col-0 plants were transformed using the *Agrobacterium*-mediated floral-dip method. Putative transformants were screened using the corresponding selection medium and verified by PCR. Positive plants were self-pollinated and selected over successive generations to obtain stable homozygous transgenic materials for subsequent experiments.

For wheat transformation, immature embryos were used as explants and transformed with *A. tumefaciens* strain EHA105 carrying pCAMBIA2300-AsTCP38-GFP using an *Agrobacterium*-mediated immature-embryo transformation procedure. Following *Agrobacterium* infection and co-cultivation, the explants underwent callus induction, selection, shoot regeneration, and rooting. Putative transgenic plants were identified using gene-specific PCR and advanced through successive generations. Five independent homozygous T3 *AsTCP38*-overexpressing wheat lines with stable transgene inheritance were obtained, and representative lines were used for subsequent physiological and biochemical analyses under low-nitrogen conditions.

### 4.5. Stress Treatment of Transgenic A. thaliana and Wheat

The transgenic and wild-type (WT) *A. thaliana* seeds were surface-disinfected with 75% ethanol (containing 0.1% Triton X-100(Sigma-Aldrich, St. Louis, MO, USA)), and then evenly sown in 1/2 MS solid medium containing 1% AGAR (pH 5.8) and placed in an artificial climate chamber (22 ± 1 °C, 16 h of light/8 h of darkness). These were cultivated vertically at 60% relative humidity for 7 days. Seedlings with consistent plant height and root development were selected and transplanted into pure vermiculite, with one plant per pot. These continued to cultivate for 21 days. From the 22nd day onward, the modified Hogland nutrient solution was irrigated every 3 days (control group: 10 mM NH_4_NO_3_; low-nitrogen group: 1.25 mM NH_4_NO_3_, with the concentrations of other elements remaining unchanged). For each genotype and nitrogen treatment, three independent biological replicates were established, with each biological replicate comprising five individual plants grown in separate pots. On the 9th day of nitrogen stress treatment, the 4th to 6th true leaves of each strain were collected. For each biological replicate, equal amounts of leaf tissue from five plants were pooled to generate one composite sample, resulting in three independently pooled biological samples (*n* = 3) for each genotype × treatment combination. Samples were immediately frozen in liquid nitrogen and stored at −80 °C until subsequent analyses.

Transgenic and WT wheat seeds were disinfected with 0.1% HgCl_2_ for 10 min, then placed on sterilized filter paper in Petri dishes, with sterile distilled water added. They were incubated in the dark at 25 °C for 7 days. Seedlings showing consistent germination were selected and transferred to a hydroponic system (Hogland complete nutrient solution, pH 6.0) with continuous aeration. The nitrogen-free modified Hoagland basal solution contained KCl (152.828 mg L^−1^), K_2_HPO_4_·3H_2_O (456.638 mg L^−1^), MgSO_4_·7H_2_O (246.480 mg L^−1^), Fe-EDTA (11.170 mg L^−1^), H_3_BO_3_ (1.546 mg L^−1^), MnSO_4_·H_2_O (0.338 mg L^−1^), CuSO_4_ (0.125 mg L^−1^), ZnSO_4_·H_2_O (0.576 mg L^−1^), and Na_2_MoO_4_ (0.102 mg L^−1^). No nitrogen-containing compound was included in the basal solution. NH_4_NO_3_ was added separately at 10 mM for the normal-nitrogen treatment and 1.25 mM for the low-nitrogen treatment. They were pre-cultivated in a light incubator (with a 25/18 °C diurnal temperature cycle and 14 h of light) for 21 days. Afterward, the modified Hogland nutrient solution was replaced (control group: 10 mM NH_4_NO_3_; low nitrogen group: 1.25 mM NH_4_NO_3_), and the nutrient solution was replenished every 3 days to maintain EC stability [5]. Wheat plants were cultivated in hydroponic containers equipped with 20 individual planting holes. For each genotype and nitrogen treatment, three independent hydroponic containers were used, with each container serving as one independent experimental unit and biological replicate. On the 9th day of treatment, a 10 cm section in the middle of the second fully developed leaf of each plant was collected from five plants in each container. Equal amounts of leaf tissue from these five plants were pooled to generate one composite sample for each biological replicate. Thus, three independently pooled biological samples (*n* = 3) were obtained for each genotype × treatment combination. The samples were immediately frozen in liquid nitrogen and stored at −80 °C in a TDE60086FV-ULTS ultra-low-temperature freezer (Thermo Fisher Scientific, Waltham, MA, USA) until subsequent analyses.

### 4.6. Assessment of Physiological Indicators in Transgenic Plants Under Low Nitrogen Stress

Three biological replicate samplings were conducted on the same parts of the leaves of transgenic and WT plants under normal- and low-nitrogen conditions. After 9 days of nitrogen treatment, the root length and fresh weight were determined. Root length was measured from the root–shoot junction to the tip of the longest root. Fresh weight was measured immediately after harvesting, after gently removing the surface moisture.

For biochemical analyses, leaf tissues were collected from the same positions of transgenic and WT plants as described above. Peroxidase (POD) and superoxide dismutase (SOD) activities were determined using commercial assay kits from Beijing Solarbio Science & Technology Co., Ltd. (BC0090 and BC5165, respectively; Beijing, China) according to the manufacturer’s instructions. CAT activity was determined using a commercial assay kit from Beijing Solarbio Science & Technology Co., Ltd. (Beijing, China) according to the manufacturer’s instructions. Glutamine synthetase (GS), nitrate reductase (NR), and starch phosphorylase (SP) activities were determined using commercial assay kits from Suzhou Comin Biotechnology Co., Ltd. (GS-1-Y, NR-1-W, and SP-1-W, respectively; Suzhou, China) according to the manufacturer’s instructions. All measurements were performed using three independent biological replicates (*n* = 3).

### 4.7. Determining of Endogenous Hormone Levels in Transgenic Plants Under Low Nitrogen Stress

Leaf samples stored at −80 °C were rapidly ground into a fine powder in a mortar pre-cooled with liquid nitrogen. Pre-cooled 80% methanol containing 1 mM butylated hydroxytoluene (BHT, Thermo Fisher Scientific, Waltham, MA, USA) was added at a solid-to-liquid ratio of 1:10 (*w*/*v*), and the samples were extracted with shaking in an ice bath for 4 h. After centrifugation at 12,000× *g* and 4 °C for 15 min, the supernatant was collected and purified using an activated C18 solid-phase extraction column (Agilent Bond Elut, 50 mg/mL, Agilent Technologies, Santa Clara, CA, USA). The column was sequentially conditioned with 3 mL methanol and 3 mL ultrapure water before sample loading. The target hormone fractions were eluted with 2 mL of methanol–ethyl acetate (1:1, *v*/*v*). The eluate was concentrated under a stream of nitrogen, reconstituted to a final volume of 200 μL with PBS buffer (pH 7.4), and stored at −20 °C in the dark until analysis.

The contents of indole-3-acetic acid (IAA), abscisic acid (ABA), salicylic acid (SA), and jasmonic acid (JA) were determined using plant hormone ELISA kits from Shanghai Enzyme-linked Biotechnology Co., Ltd., Shanghai, China. Briefly, standards and samples (50 μL per well) were added to the pre-coated microplates and incubated at 37 °C for 1 h. Biotin-labeled antibodies, HRP-labeled streptavidin, and TMB substrate were subsequently added according to the manufacturer’s instructions. Absorbance was measured at 450 nm using a BioTek Synergy H1 microplate reader (BioTek, Santa Clara, CA, USA). Three technical replicates were performed for each biological sample. Hormone concentrations were calculated from the corresponding standard curves, for which the coefficients of determination (R^2^) were >0.99.

### 4.8. Yeast Two-Hybrid (Y2H) Screening and Bimolecular- Fluorescence Complementation (BiFC) Assay

For Y2H screening, mature leaves of the low-nitrogen-tolerant *Avena sativa* cultivar Jiayan No. 2 were collected at 0 and 24 h after low-nitrogen treatment (1.25 mM NH_4_NO_3_) and used to construct a low-nitrogen-responsive cDNA library in the pGADT7-DEST prey vector (OE Biotech Co., Ltd., Shanghai, China). A truncated sequence of AsTCP38 was cloned into the pGBKT7 bait vector to generate pGBKT7-AsTCP38 and introduced into Y2HGold yeast cells using the PEG/LiAc method. The pGBKT7-53/pGADT7-T and pGBKT7-Lam/pGADT7-T combinations were used as positive and negative controls, respectively, and the empty pGBKT7 vector was included for bait autoactivation assessment. Library screening was initially performed on SD/-Trp/-Leu/X-α-Gal/AbA medium, followed by stringent selection on SD/-Trp/-Leu/-His/-Ade/X-α-Gal/AbA medium at 30 °C for 3–5 d. Positive blue colonies were recovered and identified by Sanger sequencing. Candidate prey plasmids were subsequently recovered and individually co-transformed with pGBKT7-AsTCP38 into Y2HGold cells for pairwise interaction verification under the same stringent selection conditions.

For the BiFC assays, AsTCP38 and the candidate interacting proteins were fused to complementary YFP fragments using the G2/G4 BiFC vector system. The corresponding empty-vector combinations, including G2 + G4, AsTCP38-G2 + G4, and G2 + candidate protein-G4, were used as negative controls. The recombinant constructs were introduced into Agrobacterium tumefaciens and transiently co-expressed in Nicotiana benthamiana leaves. Agrobacterial suspensions were adjusted to an OD600 of approximately 0.4 before infiltration. After infiltration, plants were maintained in darkness overnight, and fluorescence was examined 2–3 d later using an FV10-ASW confocal laser-scanning microscope. YFP fluorescence was excited at 515 nm, and chlorophyll autofluorescence was monitored at 488 nm. Reconstituted YFP fluorescence was considered indicative of protein interaction. All BiFC assays were independently repeated three times with consistent results.

### 4.9. RNA Extraction and Quantitative Real-Time PCR

Transgenic and WT *A. thaliana* and wheat plants were subjected to normal-nitrogen (10 mM NH_4_NO_3_) and low-nitrogen (1.25 mM NH_4_NO_3_) treatments. Leaf tissues were collected at 0, 3, 6, and 9 d after initiation of the nitrogen treatments and immediately stored at −80 °C until RNA extraction. For each genotype, nitrogen treatment, and sampling time, three independent biological replicates were collected.

Total RNA was extracted from leaf tissues using the TaKaRa MiniBEST Plant RNA Extraction Kit and reverse-transcribed into cDNA for qRT-PCR analysis. Gene-specific primers for AsTCP38 (AsTCP38-F/AsTCP38-R) were used, with *GAPDH* as the internal reference gene (*GAPDH*-F/*GAPDH*-R; Supplementary Table S1). qRT-PCR was performed using an ABI StepOnePlus Real-Time PCR System. Each 20 μL reaction contained 10 μL of 2× SYBR Premix Ex Taq, 0.4 μL of gene-specific primers (10 μmol L^−1^), 1 μL of cDNA template, 0.4 μL of ROX, and 7.8 μL of ddH_2_O. The amplification program consisted of 95 °C for 30 s, followed by 40 cycles of 95 °C for 5 s and 60 °C for 34 s. Melting-curve analysis was performed at 95 °C for 15 s, 60 °C for 1 min, and 95 °C for 15 s. Three technical replicates were performed for each biological replicate. Relative AsTCP38 expression was calculated using the 2^−ΔΔCt^ method.

### 4.10. Data Processing and Statistical Analysis

Data are presented as the mean ± standard error (SE) of three independent biological replicates (*n* = 3), and error bars in the figures represent SE. Data were organized using Microsoft Excel 2020 (Microsoft, Redmond, WA, USA). Statistical differences were analyzed using two-way analysis of variance (two-way ANOVA), followed by Duncan’s multiple range test for post hoc multiple comparisons. Differences were considered statistically significant at *p* < 0.05. Statistical analyses and graphical presentation were performed using GraphPad Prism 10 (GraphPad Software, Boston, MA, USA).

## 5. Conclusions

This study characterized AsTCP38, a nuclear TCP transcription factor from *Avena sativa*, and examined its responses and potential function under nitrogen limitation. Heterologous expression of *AsTCP38* in *Arabidopsis* and wheat was associated with greater root growth and fresh weight under the low-nitrogen conditions tested, together with changes in antioxidant-enzyme activities, nitrogen-metabolism-related enzyme activities, and hormone levels. Y2H and BiFC assays identified AsCYTB, AsRBM24, and AsNADP-ME as candidate interacting proteins. Taken together, the results identify *AsTCP38* as a promising candidate for the further study of low-nitrogen responses. Validation in *Avena sativa* and the analysis of direct downstream targets will help determine how these molecular and physiological responses contribute to *Avena sativa* improvement.

## Figures and Tables

**Table 1:** Physicochemical property analysis of the AsTCP38 transcription factor in *Avena sativa*.

Gene Name	ID	Chromosome Location	Amino Acid Length	PI	Molecular Weight	Subcellular Localization
*AsTCP38*	*AVESA.00001b.r3.6Dg0000536.2*	6D	405	9.38	40,743.01	Nucleus

**Table 2:** Major cis-acting elements in the *AsTCP38* gene promoter region.

Cis-Acting Element	Sequence	Function	Number
G-box	CACGTG	light responsiveness	6
CGTCA-motif	CGTCA	MeJA-responsiveness	1
P-box	CCTTTTG	gibberellin-responsive element	3
TCT-motif	TCTTAC	part of a light responsive element	1
ABRE	CACGTG	abscisic acid responsiveness	4
I-box	GGATAAGGTG	part of a light responsive element	2
GT1-motif	GGTTAA	light responsive element	1
TCA-element	CCATCTTTTT	salicylic acid responsiveness	1
ARE	AAACCA	anaerobic induction	1
MRE	AACCTAA	light responsiveness	1
GATA-motif	AAGGATAAGG	part of a light responsive element	3
TCCC-motif	TCTCCCT	part of a light responsive element	2
TATC-box	TATCCCA	gibberellin-responsiveness	1

## Data Availability

The original contributions presented in this study are included in the article/Supplementary Material. Further inquiries can be directed to the corresponding author.

## Supplementary Materials

The following supporting information can be downloaded at: https://www.mdpi.com/article/10.3390/ijms27177822/s1.

## Abbreviations

ABA	Abscisic acid
ABRE	ABA-responsive element
AsTCP38	*Avena sativa* TCP transcription factor 38
BiFC	Bimolecular fluorescence complementation
CAT	Catalase
cDNA	Complementary DNA
CDS	Coding sequence
CFU	Colony-forming unit
DNA	Deoxyribonucleic acid
DRE	Dehydration-responsive element
ELISA	Enzyme-linked immunosorbent assay
GA	Gibberellin
GFP	Green fluorescent protein
GS	Glutamine synthetase
IAA	Indole-3-acetic acid
JA	Jasmonic acid
LN	Low nitrogen
MDA	Malondialdehyde
MS	Murashige and Skoog medium
NCBI	National Center for Biotechnology Information
NR	Nitrate reductase
OE	Overexpression
ORF	Open reading frame
PCR	Polymerase chain reaction
PCF	Proliferating cell factor
PEG	Polyethylene glycol
POD	Peroxidase
qPCR	Quantitative PCR
qRT-PCR	Quantitative real-time PCR
RNA	Ribonucleic acid
ROS	Reactive oxygen species
SA	Salicylic acid
SD	Synthetic dropout medium
SOD	Superoxide dismutase
SP	Starch phosphorylase
TCP	Teosinte branched1/Cycloidea/Proliferating cell factor
WT	Wild type
Y2H	Yeast two-hybrid
YFP	Yellow fluorescent protein
